# Supplementary material for: Characterization of five environmental phages infecting Escherichia coli K-12 isolated during a phage biology training course
Source: Microbiol Spectr. 2025 Nov 6;13(12):e02274-25. doi: 10.1128/spectrum.02274-25 (PMC12671086; doi:10.1128/spectrum.02274-25)
Supplement: Figure S3 — Visualization of LuPh5 and the nine most similar phages. [file spectrum.02274-25-s0004.pdf]

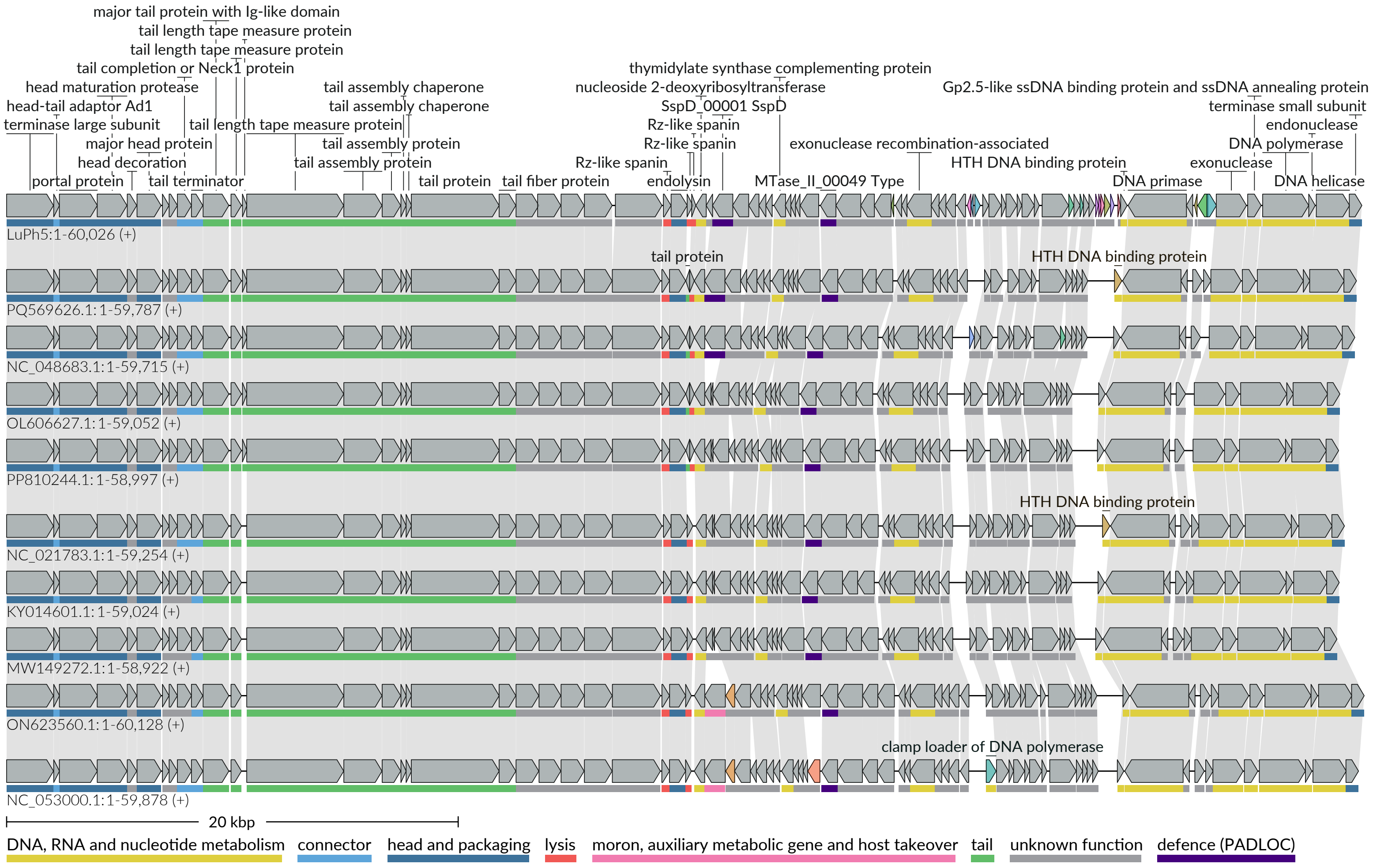

Fig. S3. Visualization of LuPh5 and the nine most similar phages, generated using LoVis4u. Conserved genes are shown in gray, while genes encoding variable protein groups are highlighted with distinct colors. Protein classifications are derived from LoVis4u analysis of all LuPh5-like phages identified via BLAST search. Functional annotations are indicated by colored lines beneath each gene, following the color code at the bottom.
