# Supplementary material for: Characterization of five environmental phages infecting Escherichia coli K-12 isolated during a phage biology training course
Source: Microbiol Spectr. 2025 Nov 6;13(12):e02274-25. doi: 10.1128/spectrum.02274-25 (PMC12671086; doi:10.1128/spectrum.02274-25)
Supplement: Figure S4 — Heatmap generated by VIRIDIC. [file spectrum.02274-25-s0005.pdf]

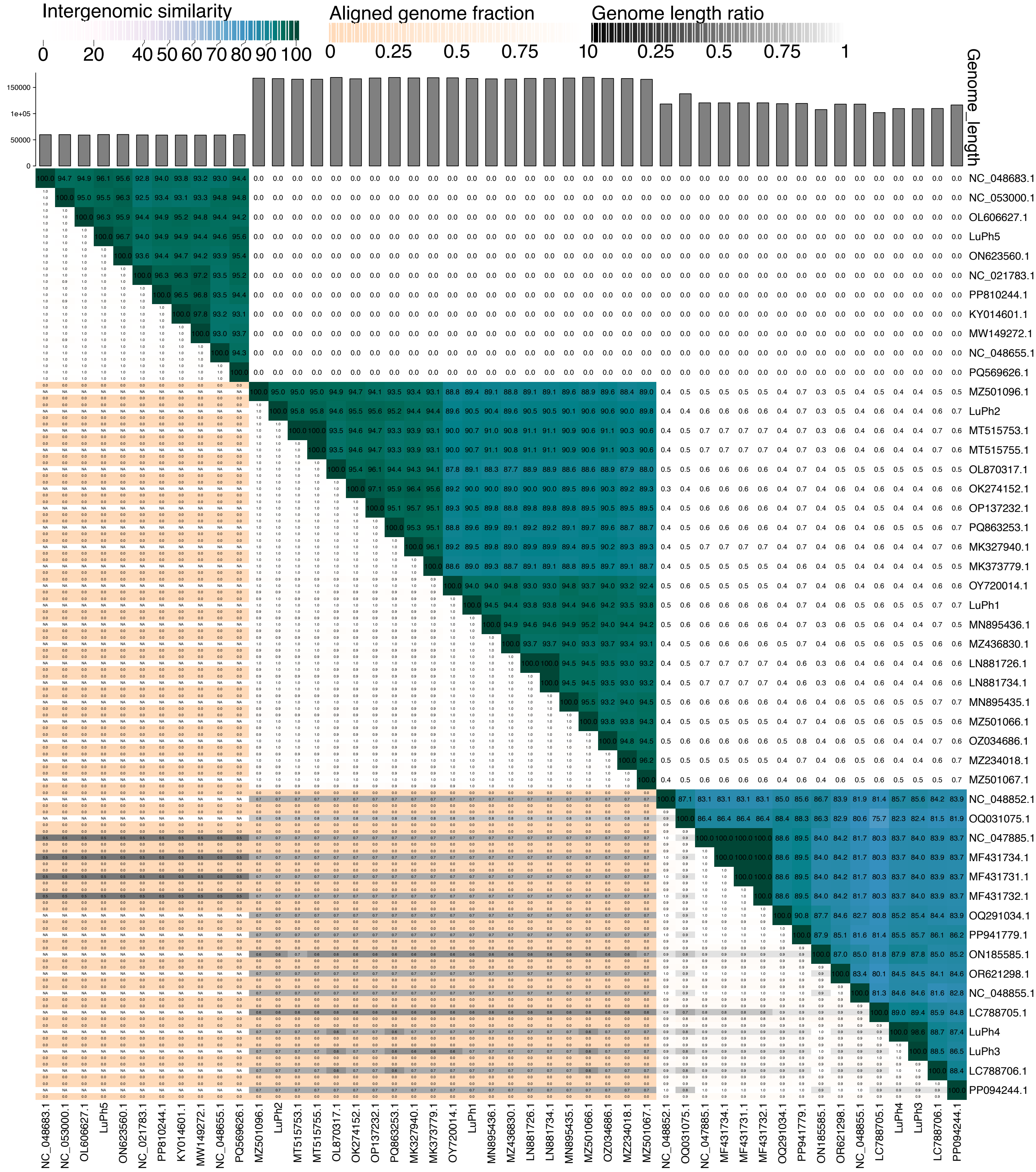

Fig. S4. Heatmap generated by VIRIDIC showing pairwise intergenomic similarities, aligned genome fractions, and genome lengths for a subset of the most similar phages to each LuPh group.
