# Supplementary material for: Characterization of five environmental phages infecting Escherichia coli K-12 isolated during a phage biology training course
Source: Microbiol Spectr. 2025 Nov 6;13(12):e02274-25. doi: 10.1128/spectrum.02274-25 (PMC12671086; doi:10.1128/spectrum.02274-25)
Supplement: Figure S5 — Plaque and motility assays. [file spectrum.02274-25-s0006.pdf]

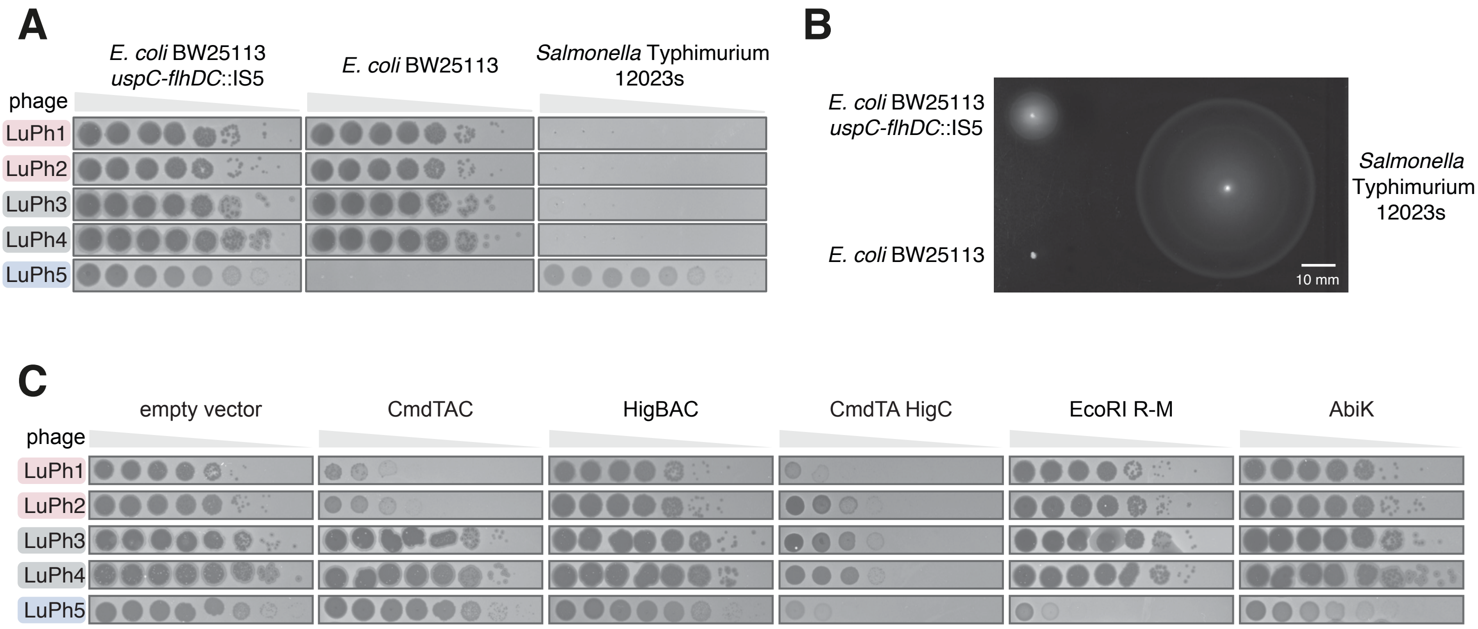

Fig. S5. Serial dilution plaque and motility assays. **(A)** Plaque assays on lawns of *E. coli* BW25113 *uspC-flhDC*::IS5 (VHB17), *E. coli* BW25113 (VHB987), and *Salmonella* Typhimurium 12023s. The panel shows 24 h incubation of the of the assay shown in Fig. 2D. **(B)** Swimming motility assay on a soft agar plate. The plate was imaged after incubation at 37°C for 8 h. **(C)** Plaques assays on lawns of *E. coli* BW25113 *uspC-flhDC*::IS5 (VHB17) harboring either an empty vector or a plasmid containing the indicated anti-phage defense system The panel shows 24 h incubation of the of the assay shown in Fig. 2F.
